# Supplementary material for: Distinct genomic routes underlie transitions to specialised symbiotic lifestyles in deep-sea annelid worms
Source: Nat Commun. 2023 May 17;14:2814. doi: 10.1038/s41467-023-38521-6 (PMC10192322; doi:10.1038/s41467-023-38521-6)
Supplement: Supplementary file 1 — Supplementary Information [file 41467_2023_38521_MOESM1_ESM.pdf]

Supplementary Information

Distinct genomic routes underlie transitions to specialised symbiotic lifestyles in deep-sea annelid worms

Index

- Supplementary Figures 1–16
- Supplementary Tables 1–7

Supplementary Figures

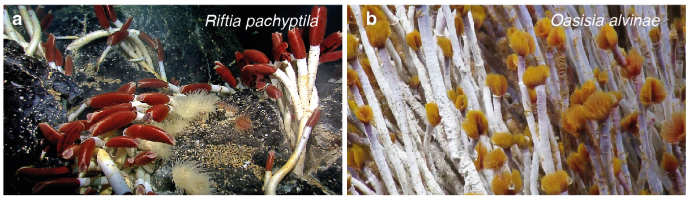

c

Species	Location	Equipment	Collection date
Osedax frankpressi	Whalefall 2893 (Ruby), Monterey Submarine Canyon, California (US)<br>Depth: 2893m	ROV Tiburon, ship R/V Western Flyer	30 November 2004
Riftia pachyptila	Matterhorn vent site, Gulf of California (Mexico)<br>Depth: 3653.5m	ROV SuBastian, ship R/V Falkor	14 November 2018
Oasisia alvinae	Matterhorn vent site, Gulf of California (Mexico)<br>Depth: 3653.5m	ROV SuBastian, ship R/V Falkor	14 November 2018

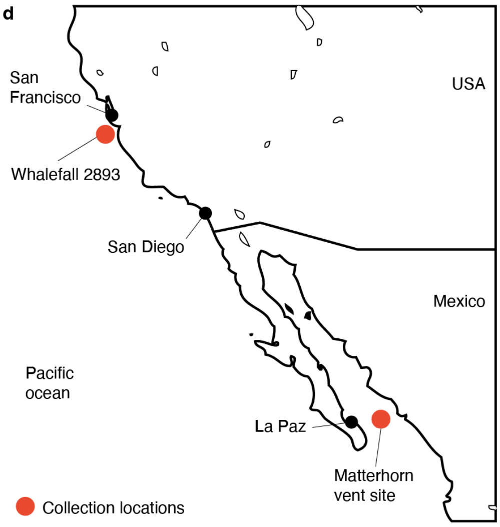

Supplementary Figure 1. Focal taxa and collection sites. (**a**, **b**) Photographs of *R. pachyptila* and *Oasisia alvinae* adult worms. Source, wikicommons. (**c**) Table indicating the location, equipment used for the collection, and collection date of the specimens used for genome sequencing for the three species of Siboglinidae herein studied. Voucher specimens are lodged at the Scripps Institution of Oceanography Benthic Invertebrate Collection. (**d**) The schematic map of southern California and northwest Mexico indicates the main cities (black dots) and collection sites (red dots).

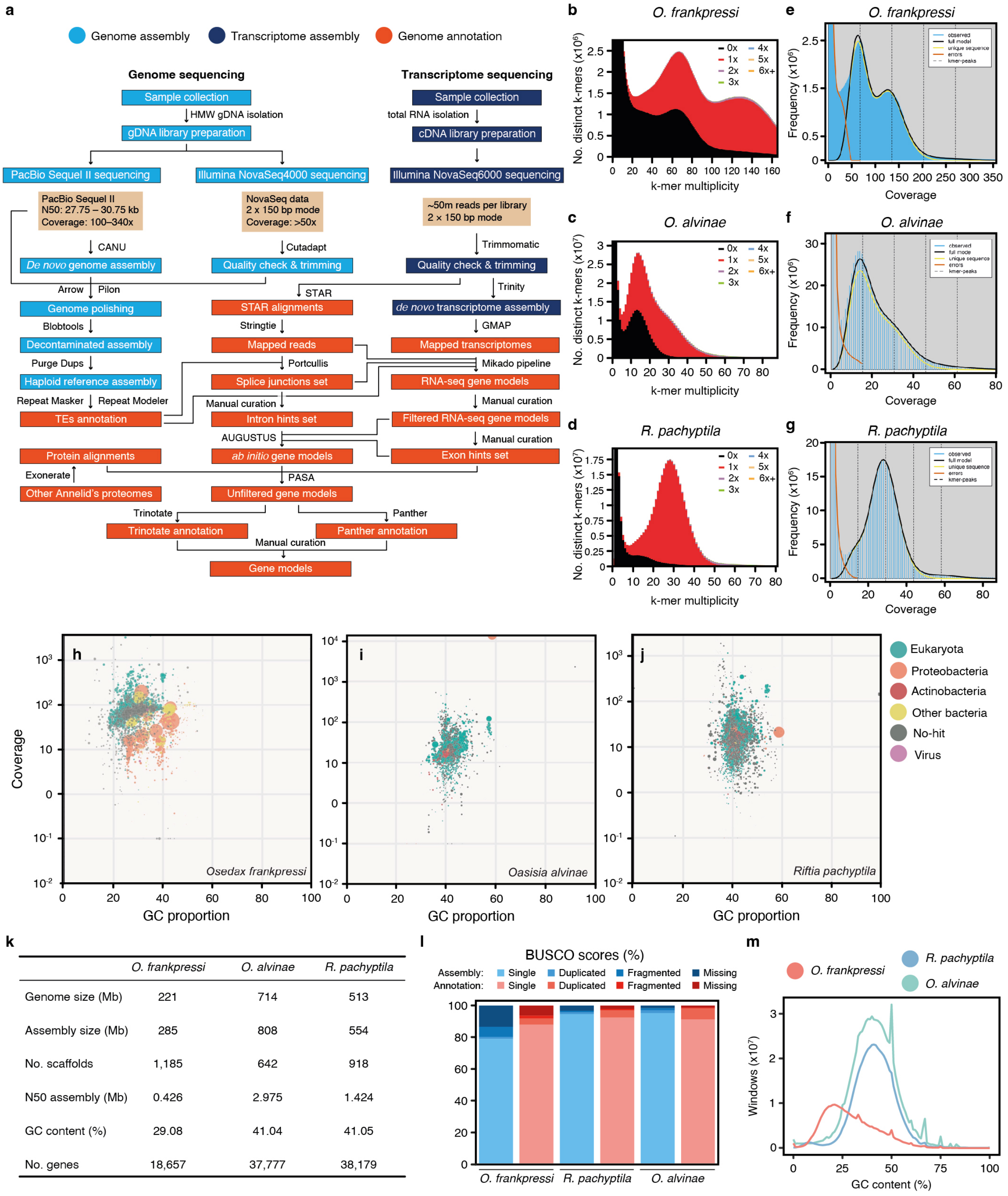

k

	O. frankpressi	*O. alvinae*	*R. pachyptila*
Genome size (Mb)	221	714	513
Assembly size (Mb)	285	808	554
No. scaffolds	1,185	642	918
N50 assembly (Mb)	0.426	2.975	1.424
GC content (%)	29.08	41.04	41.05
No. genes	18,657	37,777	38,179

Supplementary Figure 2. The genomes of *O. frankpressi*, *Oasisia alvinae* and *R. pachyptila*. (**a**) Schematic diagram outlining the strategy followed to sequence and assemble the genomes (light blue boxes), transcriptomes (dark blue boxes) and functionally annotate the genomes (red boxes) of the three focal species of this study. See Materials and Methods for details. (**b**–**d**) *k*-mer distribution plots indicating that the genome assemblies for *O.*

frankpressi, *Oasisia alvinae* and *R. pachyptila* are largely de-haploidised. (**e**–**g**) GenomeScope 2.0 profiles showing *k*-mer-based genome size estimations for the three studied species. (**h**–**j**) Blobtools plots for *O. frankpressi*, *Oasisia alvinae* and *R. pachyptila* show eukaryotic contigs in green and prokaryotic light yellow and red. Consistent with the initial biological samples used for genomic extraction (entire animal for *O. frankpressi* and trunk piece including the trophosome for *Oasisia alvinae*), the genomes of those two annelids include their endosymbionts and associated epibiota. (**k**) Table with basic assembly statistics for the three species of study. (**l**) Bar plot indicating the proportions of BUSCO genes in the assemblies (blue bars) and annotations (red bars) of the three focal species. Note how gene annotation improves BUSCO scores in *O. frankpressi*. (**m**) Line plots of GC content in the three Siboglinidae of study.

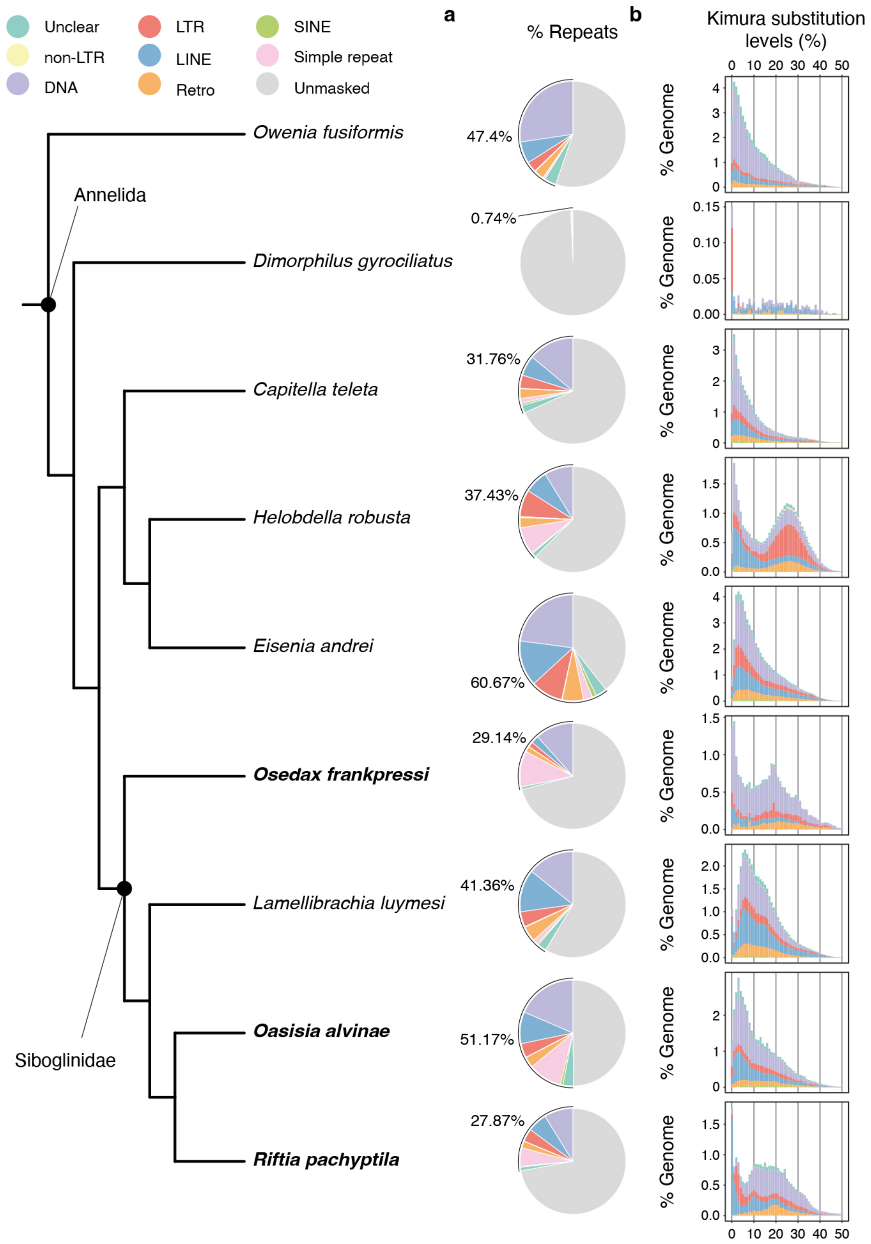

Supplementary Figure 3. The annelid repetitive landscape. (**a**) Pie charts showing proportions of repetitive elements in nine annelid genomes under a consensus tree topology (on the left). The genomes of the species sequenced in this study are highlighted in boldface. Despite having a smaller genome, *O. frankpressi* has relatively similar repeat fractions than other Siboglinidae (e.g., *R. pachyptila*) and asymbiotic annelids (e.g., *C. teleta*). (**b**) The distribution plots on the figure's left show the Kimura substitution levels of repeat elements and their proportion in the genome for the same nine annelid species. As observed in other Siboglinidae (e.g., *R. pachyptila*), *O. frankpressi* experienced a past expansion of

transposable elements (Kimura substitution levels from 10% to 30%), mainly involving DNA transposons.

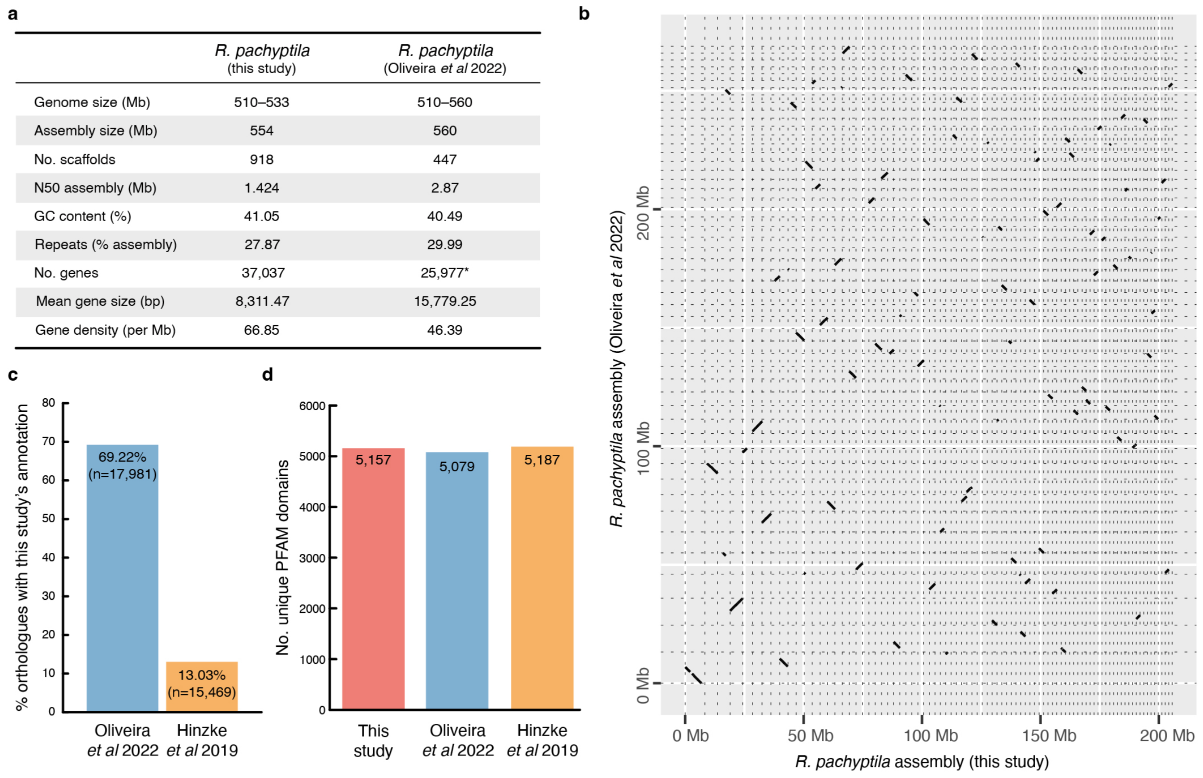

	R. pachyptila (this study)	*R. pachyptila* (Oliveira *et al* 2022)
Genome size (Mb)	510–533	510–560
Assembly size (Mb)	554	560
No. scaffolds	918	447
N50 assembly (Mb)	1.424	2.87
GC content (%)	41.05	40.49
Repeats (% assembly)	27.87	29.99
No. genes	37,037	25,977*
Mean gene size (bp)	8,311.47	15,779.25
Gene density (per Mb)	66.85	46.39

Supplementary Figure 4. Comparison of the different genomic and transcriptomic resources for *R. pachyptila*. (**a**) Table with general statistics of the genome assemblies and annotations reported in this study and Oliveira *et al*. REF10 (**b**) Dot-plot representation of the similarity between the assemblies for *R. pachyptila* reported in this study and Oliveira *et al*. REF10. Only the half, most contiguous assembly is shown. Overall, both assemblies are highly similar. (**c**) Gene-wise comparison between existing genome annotations and the transcriptomic resources in Hinzke *et al.* REF12. Nearly 70% of the genes (17,981) in Oliveira *et al.* REF10 have a one-to-one ortholog in our annotation based on reciprocal best BLAST hit. A relatively similar number of contigs (15,469) from Hinzke *et al.* REF12 have a one-to-one ortholog with our assembly. As expected, given the fragmented nature of a transcriptomic resource, these represent a much lower percentage of the total non-redundant transcriptome (13.03%). (**d**) However, the three available resources have similar unique PFAM motifs, suggesting that they all represent the diversity of proteins in *R. pachyptila*.

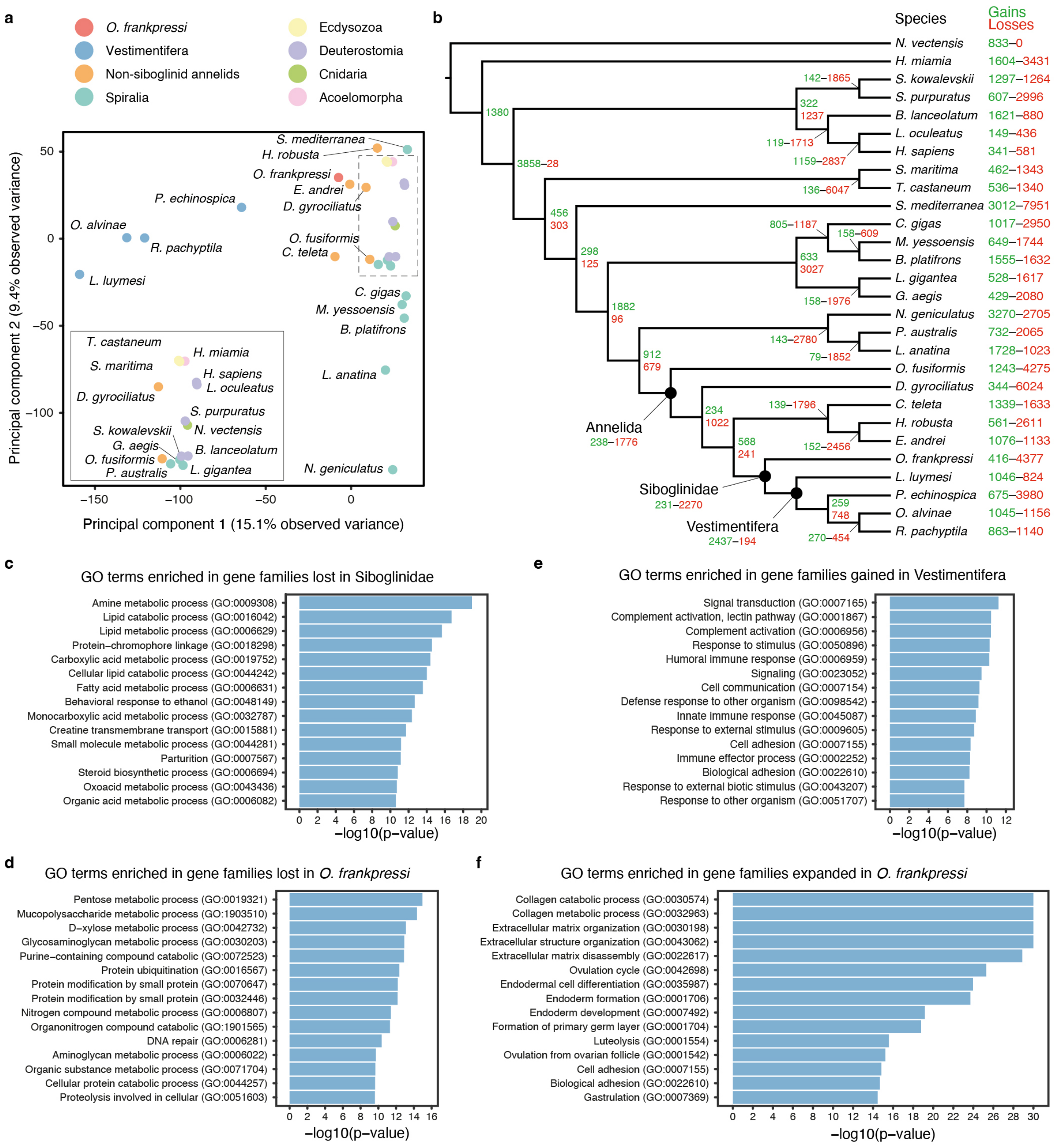

Supplementary Figure 5. The evolution of the gene repertoire of Siboglinidae. (a) Principal component analysis of 28 highly complete metazoan genomes, including all available Siboglinidae and two symbiotic molluscs (*B. platifrons* and *G. aegis*). While the symbiotic molluscan species are like their asymbiotic relatives, Vestimentifera and *O. frankpressi* are markedly different from asymbiotic annelids with slow rates of molecular evolution, such as *Owenia fusiformis* and *C. teleta*. The area squared by a dashed line in the top right corner is amplified in the bottom left corner to improve taxa identification. (**b**)

Patterns of gene family gain (green) and losses (red) for those 28 metazoan taxa under a consensus tree topology. (**c**–**f**) Bar plots showing the 15 most enriched gene ontology (GO) terms of the Biological Process class in gene families lost in Siboglinidae (**c**) and *O. frankpressi* (**d**), gained in Vestimentifera (**e**) and expanded in *O. frankpressi* (**f**). Most of the losses in Siboglinidae are enriched in GO terms for cellular metabolism. In contrast, those gained in Vestimentifera (as per *R. pachyptila*) are enriched in signal transduction, immunity, adhesion, and response to stimuli. *Osedax frankpressi* has lost more gene families enriched in GO terms related to metabolism (mostly carbohydrate) and expanded gene families involved in collagen degradation and extracellular matrix remodelling. In (**c**–**f**), *p*-values were derived from upper-tail Fisher's exact tests.

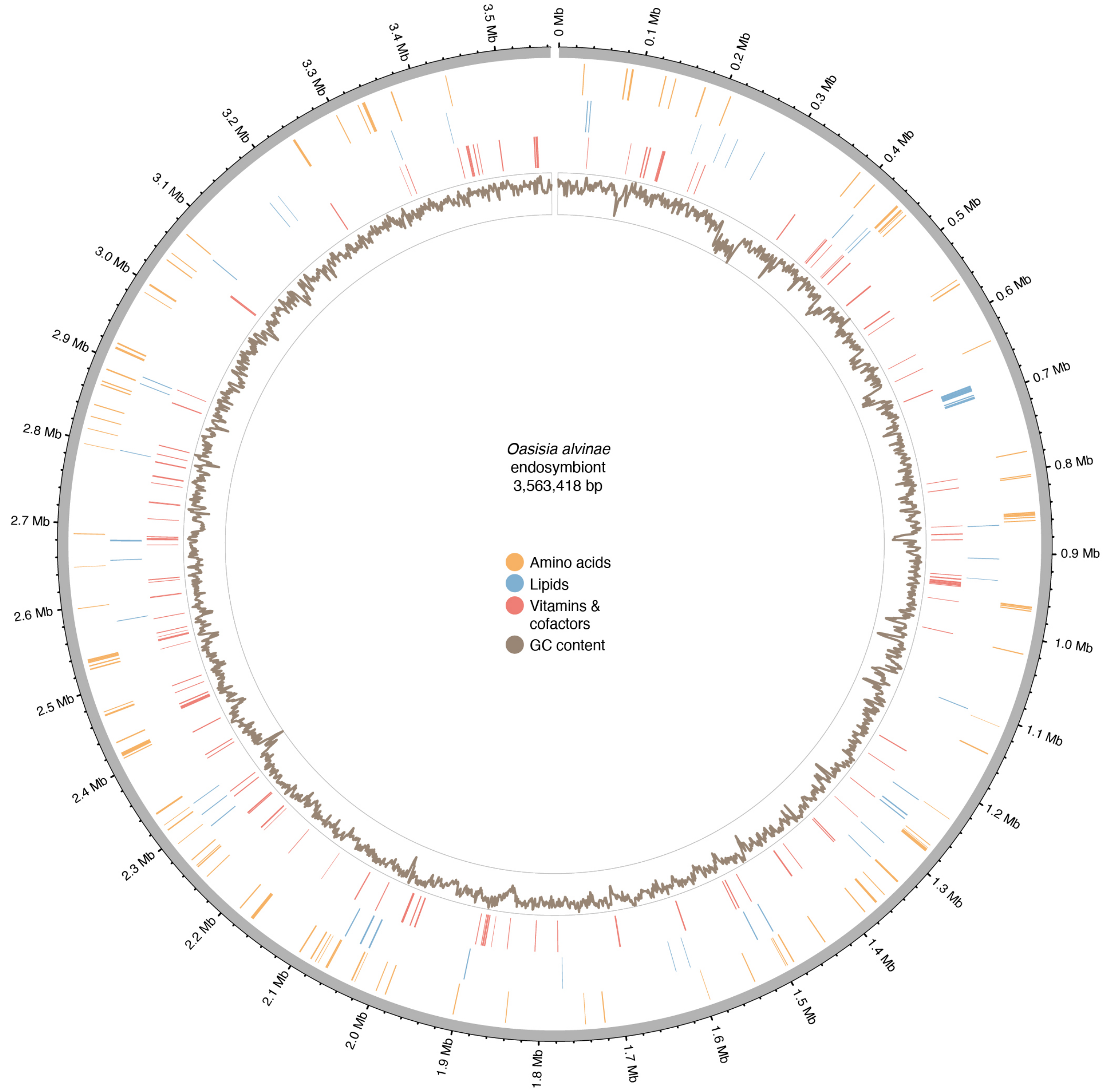

Supplementary Figure 6. *Oasisia alvinae* endosymbiont. Circular schematic representation of the genome of *Oasisia alvinae* endosymbiont assembled into a single contig. The plot shows the genomic location of genes involved in amino acid, lipid, and vitamin/cofactor metabolism (in orange, blue and red, respectively) and the GC content (inner circle; brown colour).

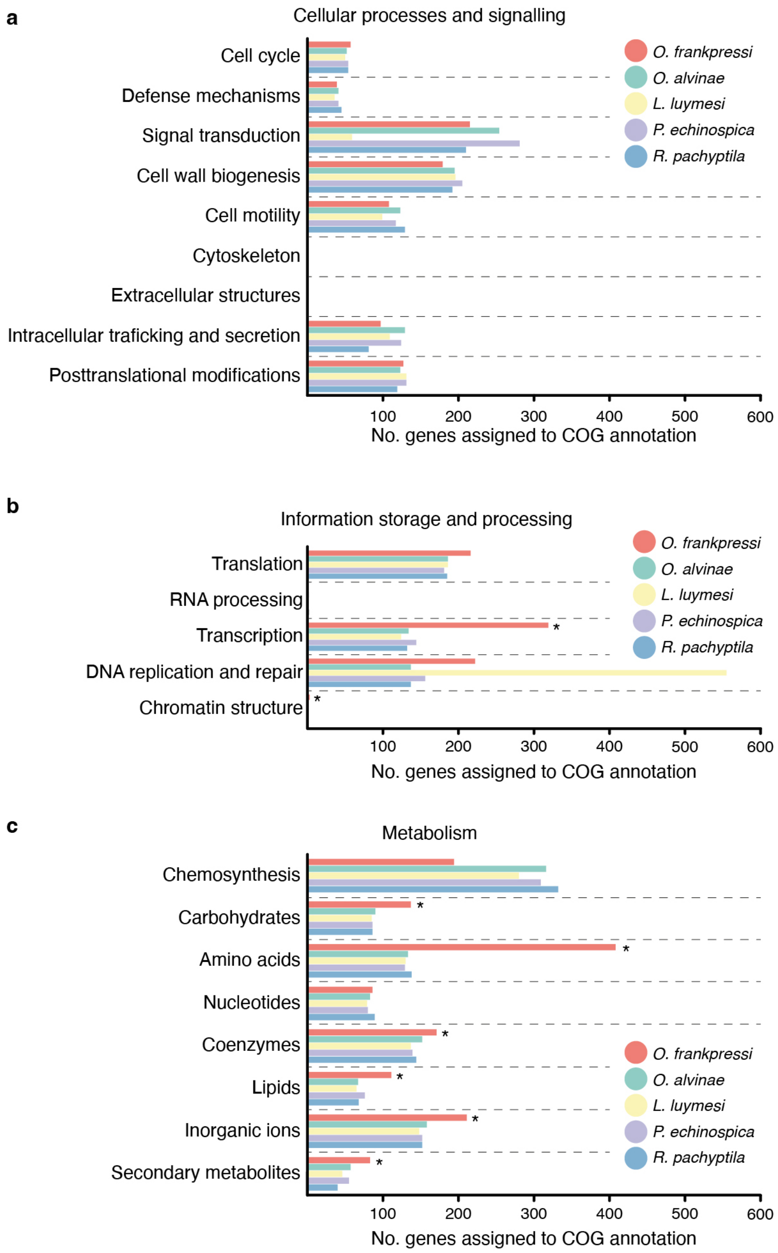

Supplementary Figure 7. The genetic toolkit of the endosymbionts of *O. frankpressi* and Vestimentifera. (**a**–**c**) Bar plots of the numbers of COG annotations involved in cellular processes and signalling (**a**), information storage and processing (**b**), and metabolism (**c**) in the genome assemblies of *Osedax*'s endosymbiont and those of the four Vestimentifera with sequenced and assembled genomes. While there are no significant differences in the repertoire of genes involved in cellular processes and signalling between the endosymbionts of *Osedax* and Vestimentifera (**a**), *Osedax*'s endosymbiont has a significantly richer

metabolic repertoire for carbohydrates, amino acids, coenzymes, lipids, inorganic ions, and secondary metabolites (**c**; significance indicated with an asterisk). However, the endosymbionts of Vestimentifera are enriched in genes involved in energy production, consistent with their chemosynthetic capacity (**c**). Genes involved in transcription and chromatin structure are also enriched in the genome of *Osedax*'s endosymbiont (**b**). Gene COG category *p*-values to test the significance of the differences between species are derived from a permutation test of the Enrichment Scores (ES) after 1,000 permutations of the Gene Set Enrichment Analysis (GSEA).

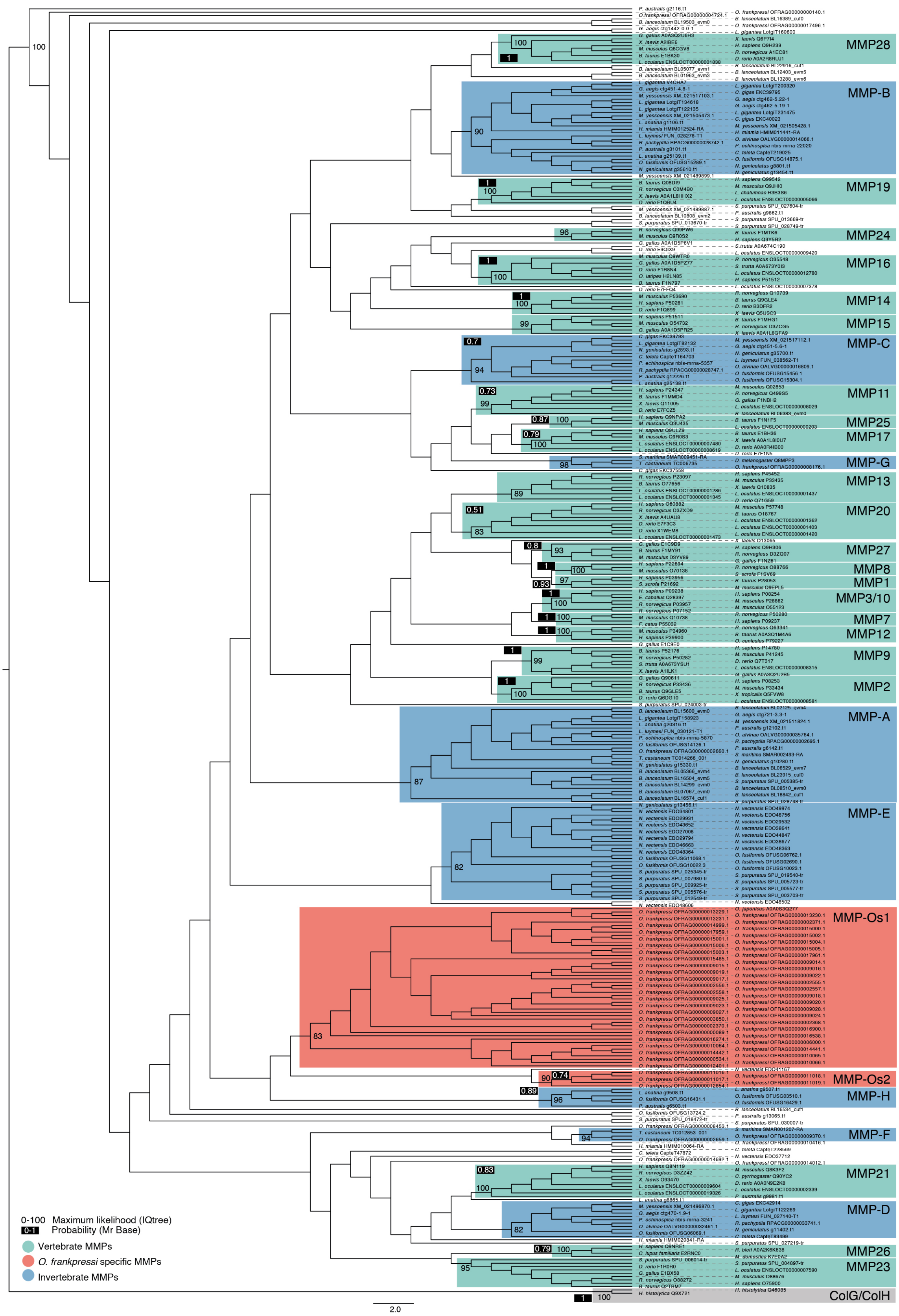

Supplementary Figure 8. Maximum likelihood phylogenetic reconstruction of animal matrix metalloproteases. Maximum likelihood consensus tree of animal matrix

metalloproteases (MMPs) using the metallopeptidase domain and ColG and ColH bacterial genes as outgroups (grey box). Light green boxes highlight vertebrate MMPs classes, invertebrate MMPs are in blue boxes, and the two independent expansions of MMPs in *Osedax* are highlighted with red boxes, showing only bootstrap values for each of the classes.

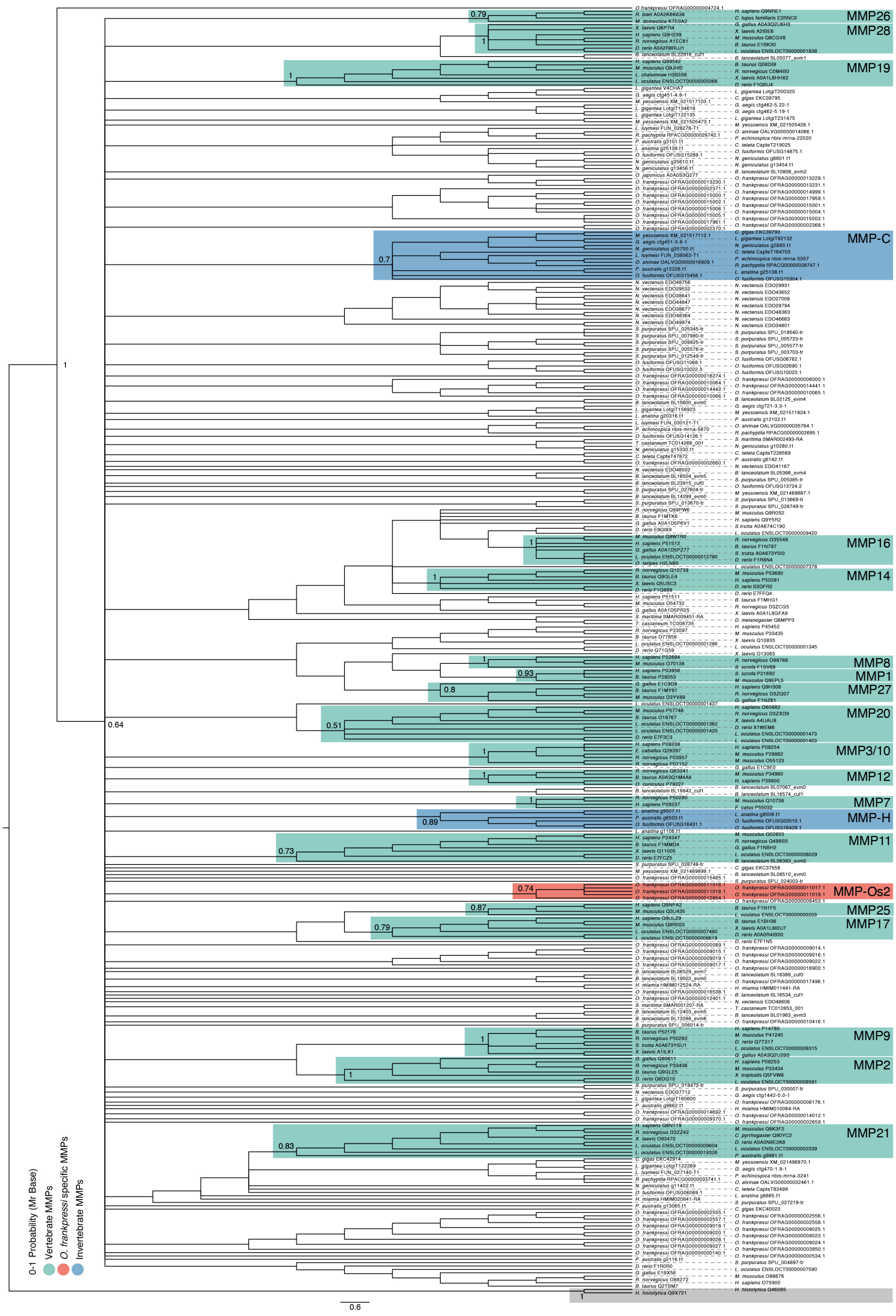

Supplementary Figure 9. Bayesian phylogenetic reconstruction of animal matrix metalloproteases. Bayesian consensus tree of animal matrix metalloproteases (MMPs) using

the metallopeptidase domain and ColG and ColH bacterial genes as outgroups (grey box). Light green boxes highlight vertebrate MMP classes, blue boxes show invertebrate MMPs, and red boxes highlight the two independent expansions of MMPs in *Osedax*. Only the posterior probabilities values for each of the classes are shown.

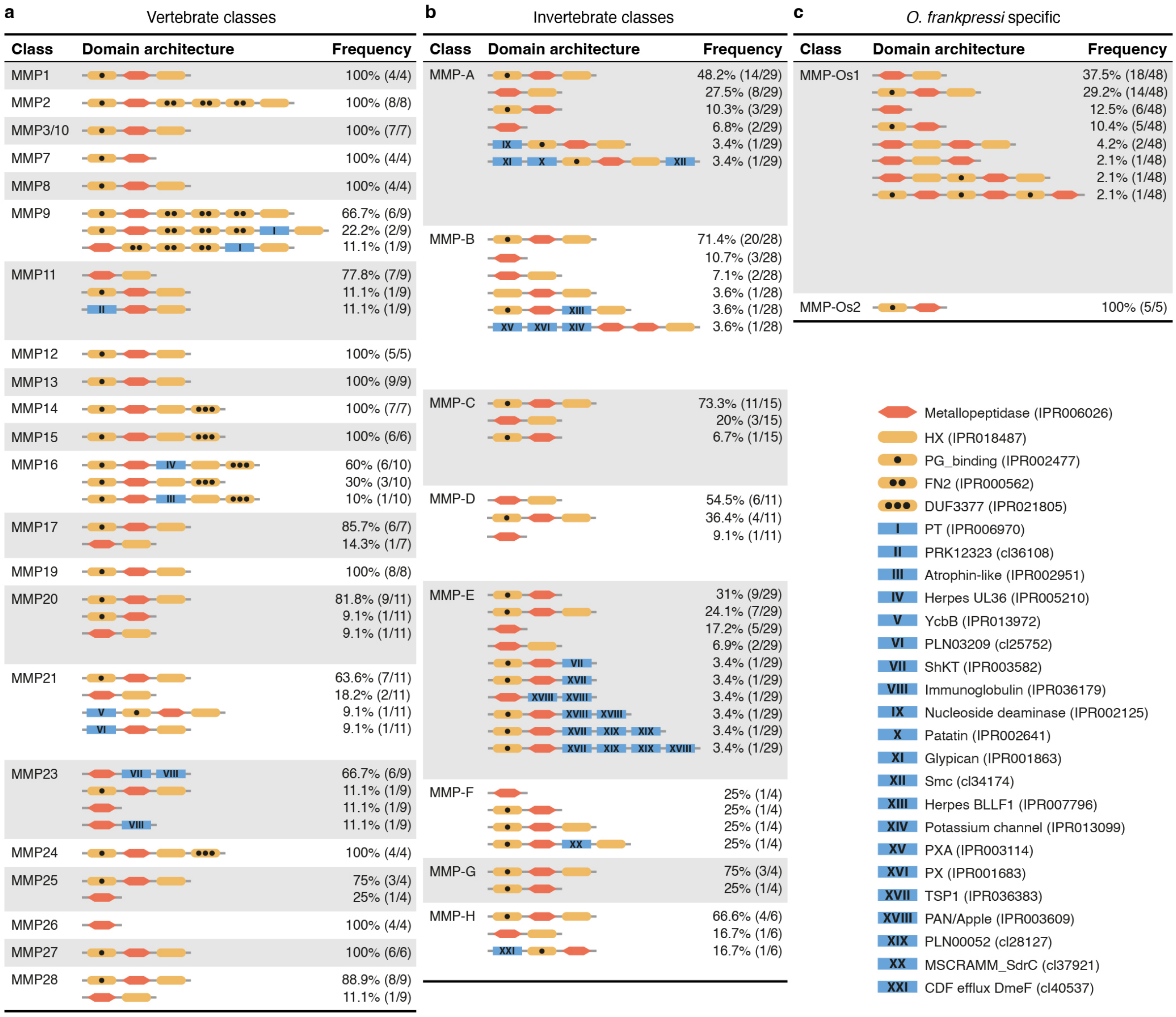

Supplementary Figure 10. The domain composition of matrix metalloproteases. (a–c) Schematic representation of the protein domain architecture of matrix metalloproteases in vertebrate (**a**), invertebrate (**b**) and *Osedax* (**c**) classes. Protein domains are coloured based on frequency (in red, the metallopeptidase domain present in all matrix metalloproteases; in yellow, relatively common domains; and in blue, domains that appear in just a reduced number of sequences or classes). The most abundant protein domain configuration for each class is shown in Figure 5B. Drawings are not to scale.

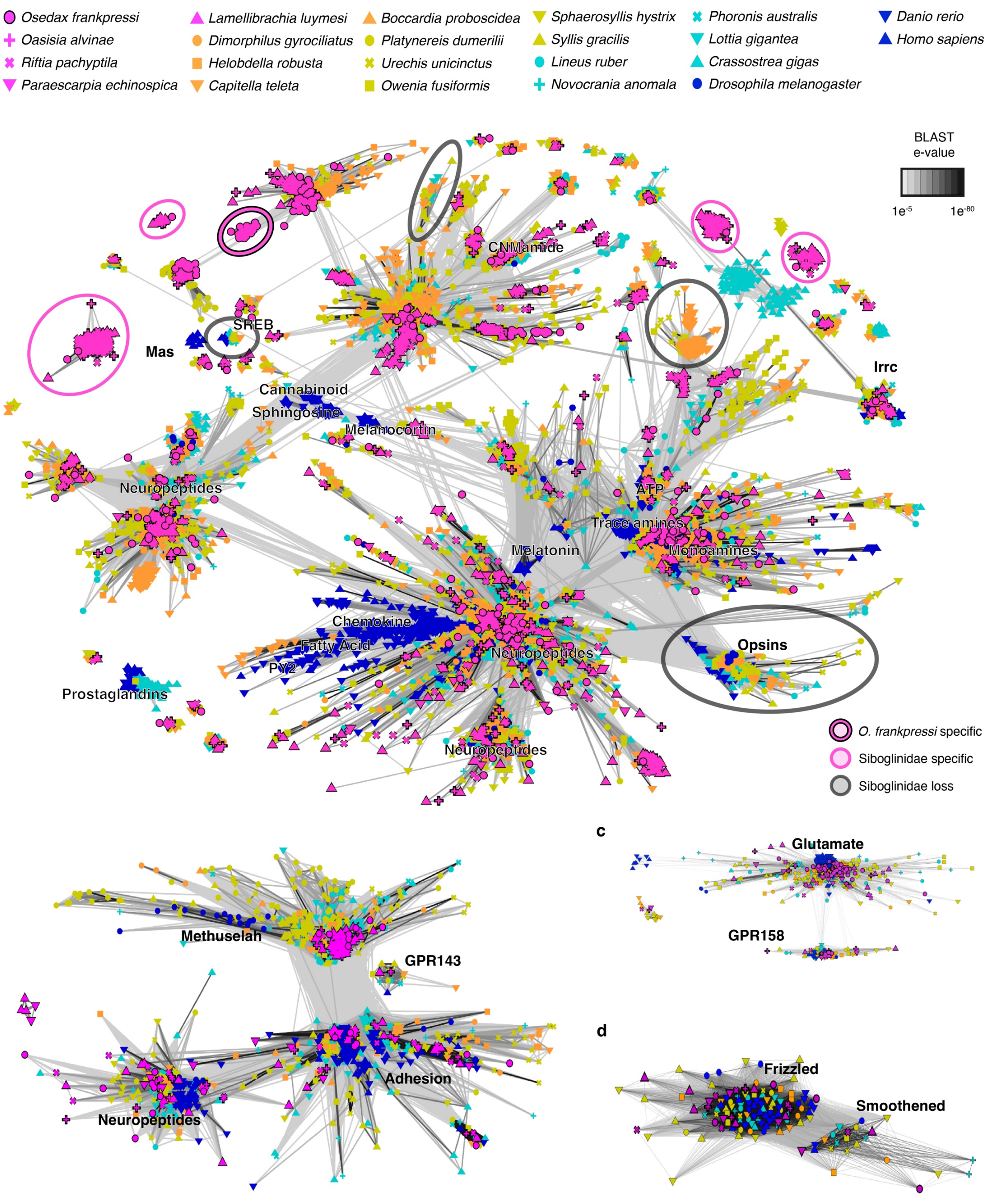

Supplementary Figure 11. The GPCR complement of Siboglinidae. (**a**–**d**) Sequence similarity cluster of G-protein coupled receptors (GPCRs) of Class A (rhodopsin-like receptors; a), B (secretin family; **b**), class C (metabotropic glutamate receptors; **c**) and class F (frizzled and smoothened receptors; **d**) in Siboglinidae (*O. frankpressi*, *Oasisia alvinae*, *R. pachyptila* and *L. luymesi*; in pink), ten other asymbiotic annelids (in orange and green) and eight other asymbiotic bilaterian lineages (spiralian species in light blue and non-spiralian

species in dark blue). Each sequence is represented by a symbol and colour according to its species of origin. Grey lines indicate similarity between these sequences based on all-to-all BLAST searches, with the intensity of grey showing the Expect value (e-value) of the search. (**a**) Siboglinidae has three clade-specific expansions (highlighted in light purple), one of them weakly related to Leucin-rich containing receptors (lrrc), and *O. frankpressi* has an additional expansion of GPCRs (light green circle in the bottom left). Siboglinidae has also lost four groups of GPCRs present in asymbiotic annelids and other bilaterians, most notably opsins and the Super Conserved Receptor Expressed in Brain (SREB) class. (**b**–**d**) All Siboglinidae have representatives of each class and orthogroups.

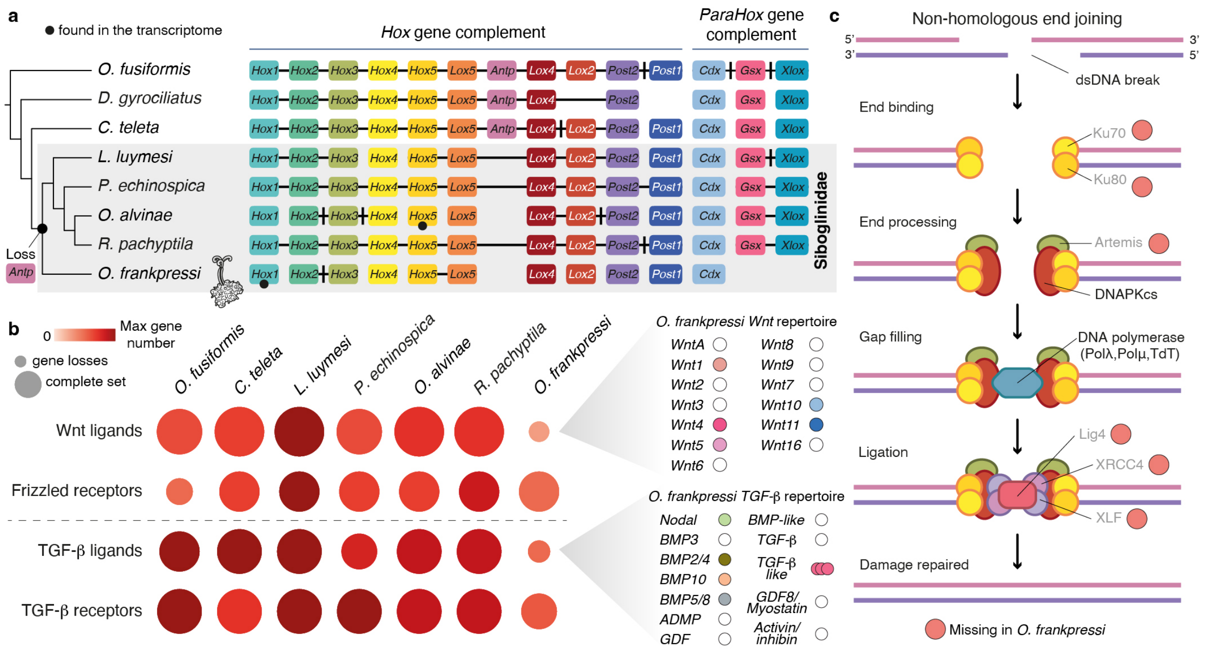

Supplementary Figure 12. The developmental and DNA repair toolkit of Siboglinidae. (**a**) Schematic representation of the *Hox* and *ParaHox* gene complements in Siboglinidae and three other asymbiotic annelid lineages. Each orthologous group is indicated with a different colour, horizontal black lines connecting boxes indicate the genomic linkage, and vertical black lines between boxes indicate interspersed genes between the corresponding *Hox*/*ParaHox* genes. Both *O. frankpressi* and Vestimentifera lack *Antennapedia* (*Antp*), and *O. frankpressi* has in addition lost *Gsx* and *Xlox*. (**b**) Plot summarising the gene number and completeness of the Wnt and TGF-β pathways (based on receptors and ligands) in Siboglinidae and two asymbiotic annelids (*Owenia fusiformis* and *C. teleta*). *Osedax frankpressi* retains the repertoire of receptors but has simplified the complement of Wnt and TGF-β ligands (right side of the panel). (**c**) Schematic representation of the non-homologous end joining DNA repair pathway, highlighting with a red circle the missing components in *O. frankpressi*. The lack of this pathway activates the microhomology-mediated end-joining repair pathway, which is present in all studied annelids, including *O. frankpressi*, and induces microdeletions that might eventually lead to genome compaction.

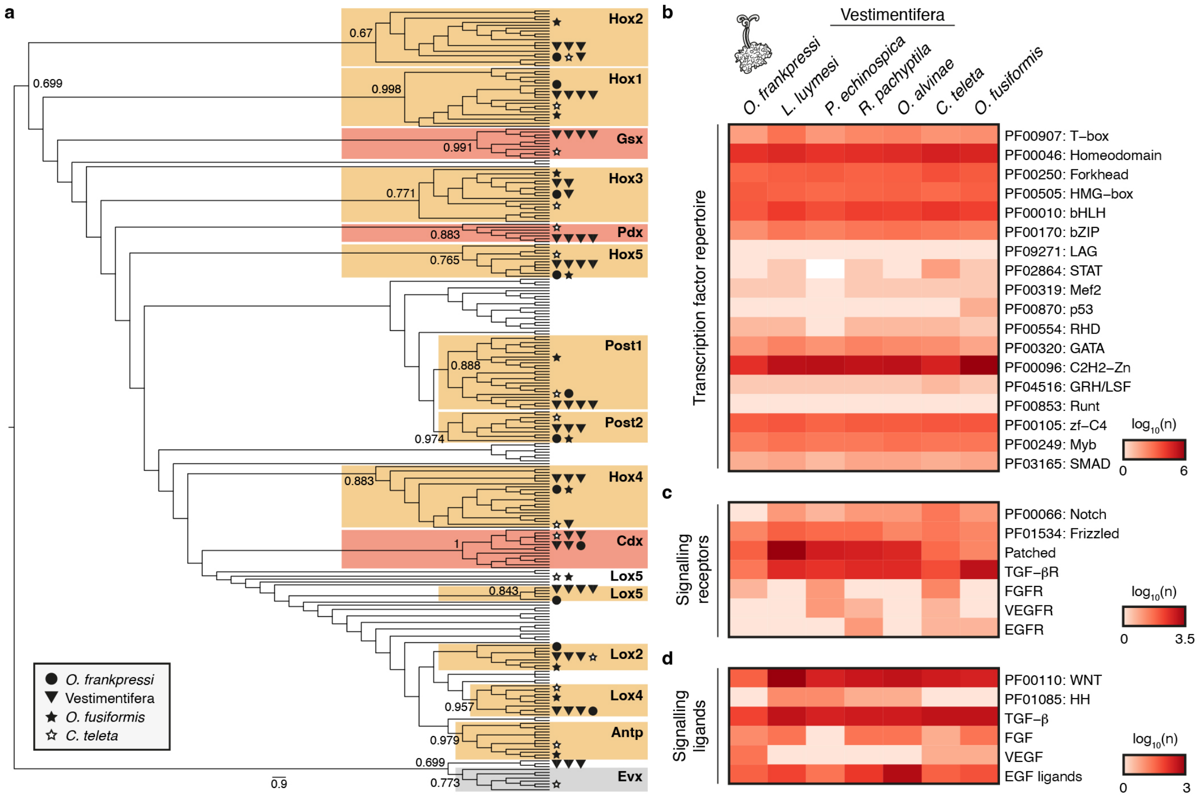

Supplementary Figure 13. The developmental toolkit and Hox complement in *O. frankpressi*. (**a**) Orthology inference cladogram of Hox and ParaHox proteins for *O. franpressi* and Vestimentifera, using Evx as outgroup and based on maximum likelihood phylogenetic reconstruction. Numbers show bootstrap support at key nodes of the tree. Symbols indicate the location of annelid sequences in the tree. (**b**–**d**) Heatmaps of the number (in $\log_{10}$ scale) of genes with transcription factor activity (**b**), signalling receptor activity (**c**) and signalling ligands (**d**). The Pfam domain and annotations used for each search are shown on the left. Compared to Vestimentifera and asymbiotic annelids, *O. frankpressi* has a reduced repertoire of Zn finger and bZIP transcription factors and Notch-containing receptors. The diversity of Wnt and TGF-b ligands is also lower in *O. frankpressi*.

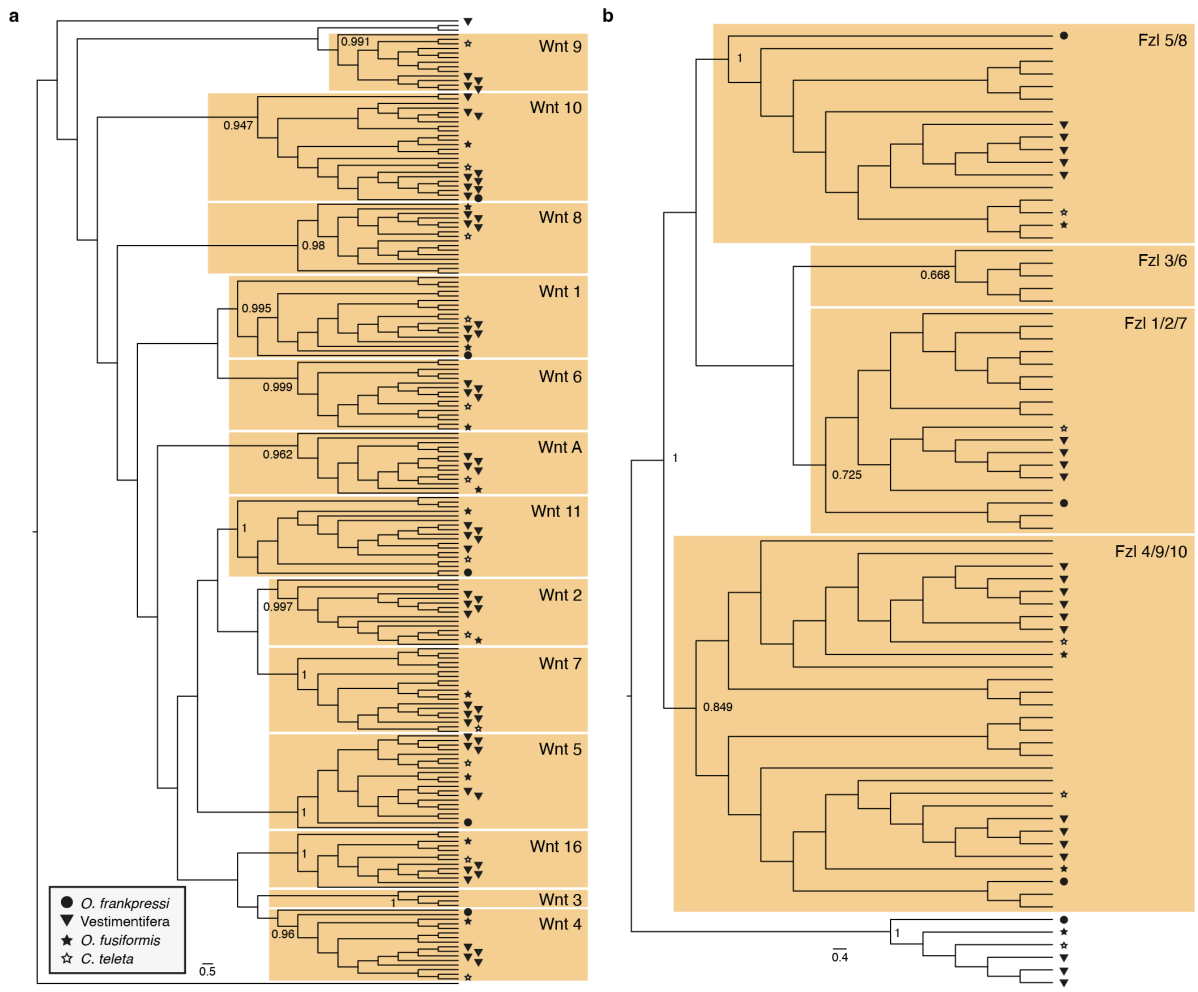

Supplementary Figure 14. The complement of ligands and receptors of the Wnt pathway in *O. frankpressi*. (**a**, **b**) Orthology inference cladograms of Wnt ligands (**a**) and receptors (**b**) highlighting each major family with a yellow box, as well as its node support (from 0 to 1). While *O. frankpressi* has an ortholog of each major group of frizzled receptors present in invertebrates (fzl5/8, fzl1/2/7 and fzl4/9/10), it has a reduced diversity of Wnt ligands, with only a copy of Wnt1, Wnt4, Wnt5, Wnt10 and Wnt11. Numbers show bootstrap support at key nodes of the tree. Symbols indicate the location of annelid sequences in the tree.

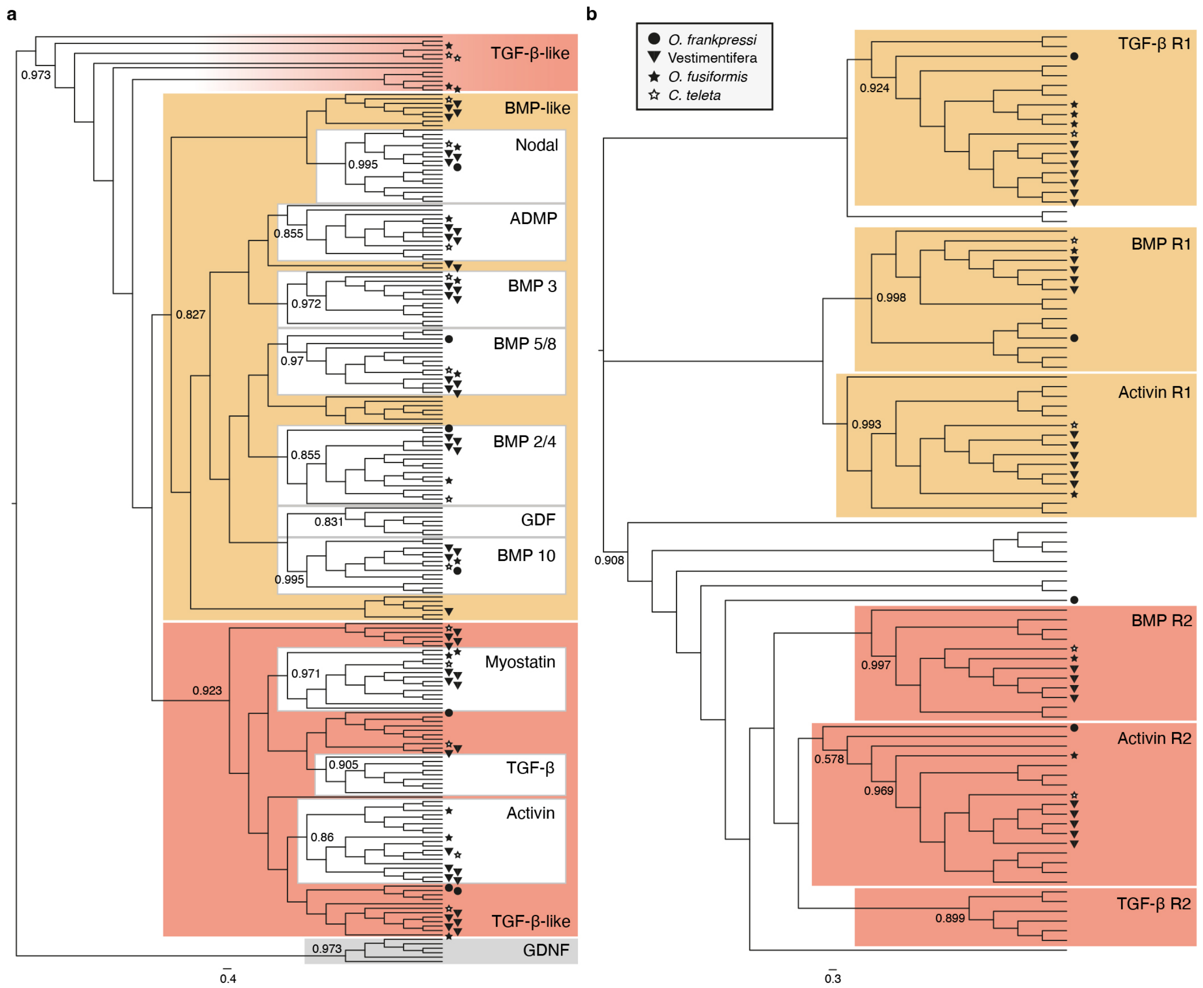

Supplementary Figure 15. The complement of ligands and receptors of the TGF-b pathway in *O. frankpressi*. (**a**, **b**) Orthology inference cladograms of TGF-β ligands (**a**) and receptors (**b**) highlighting each major family with coloured boxes (yellow, BMP type; red, TGF-β type), as well as the node support for each major clade (from 0 to 1). *Osedax frankpressi* has three TGF-β like ligands and BMP2/4, BMP5/8, BMP10 and Nodal ligands (**a**), and receptors of type 1 and type 2 for BMP and TGF-β like ligands (**b**). Numbers show bootstrap support at key nodes of the tree. Symbols indicate the location of annelid sequences in the tree.

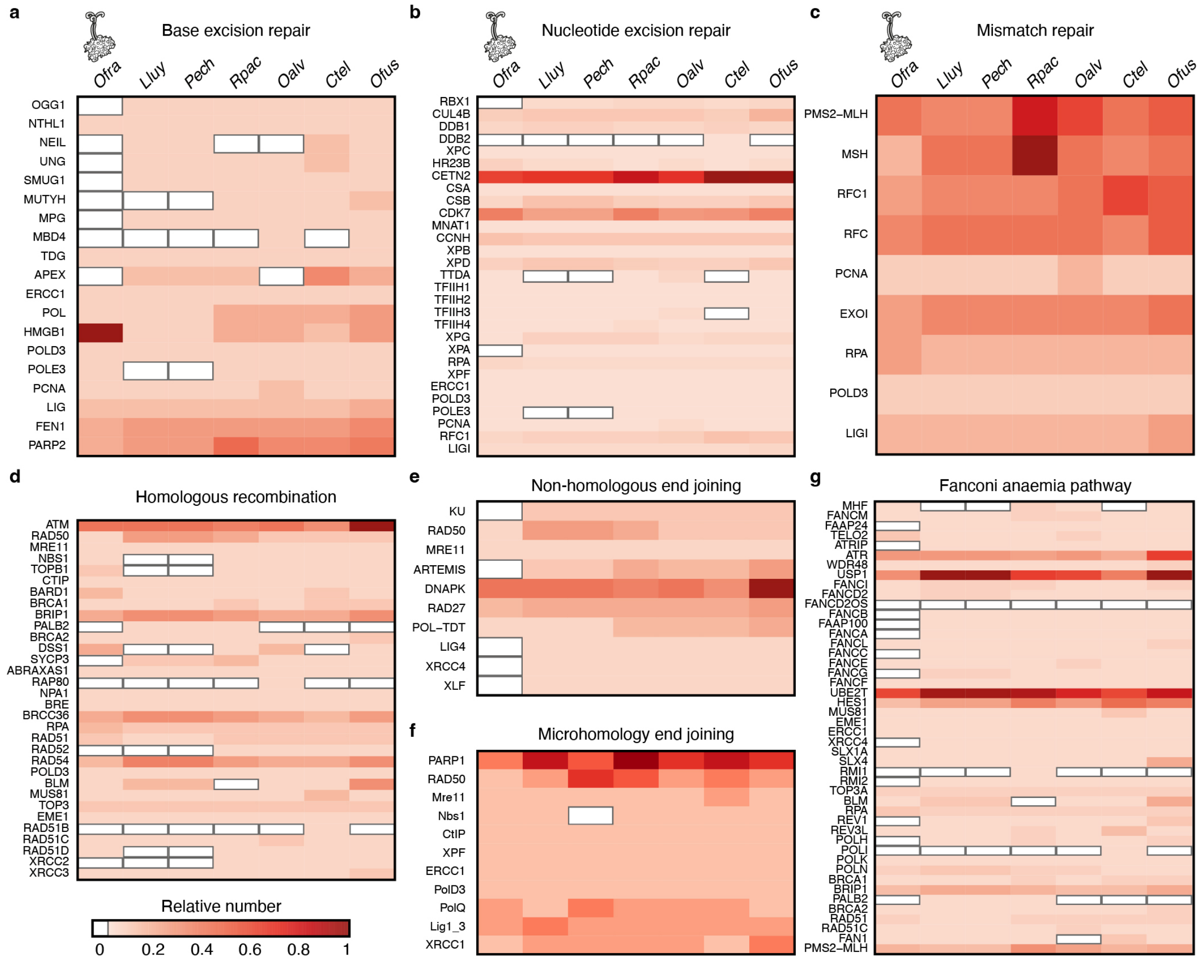

Supplementary Figure 16. The DNA repair gene complement in *Osedax* and Vestimentifera. (**a**–**g**) Heatmaps for the presence/absence and the number of orthologs for each gene and major pathway involved in the repair of lesions in the DNA based on PANTHER annotations: base excision repair (**a**), nucleotide excision repair (**b**), mismatch repair (**c**) homologous recombination (**d**), non-homologous end joining (**e**), microhomology end joining (**f**) and the Fanconi anaemia pathway (**g**). *Osedax frankpressi* (*Ofra*) shows more gene losses than other Vestimentifera (*L. luymesi*, *Lluy*; *P. echinospica*, *Pech*; *R. pachyptila*, *Rpac*; and *Oasisia alvinae*, *Oalv*) and asymbiotic annelids (*C. teleta*, *Ctel*; and *Owenia fusiformis*, *Ofus*) in the base excision repair, non-homologous end joining and Fanconi anaemia pathways. The potential lack of fully functioning DNA repair pathways might favour converting to AT nucleotides and activating the microhomology end-joining pathway, which can induce microdeletions and genome reduction. Heatmaps show relative values, with

1 representing the maximum number of genes per gene family in the given set of species. A white rectangle indicates gene loss. See Supplementary Table 26 for exact numbers and exact PANTHER ID numbers.

Supplementary Tables

Supplementary Table 1. List of genomic and transcriptomic resources per species

Species	Source	Material	Platform	reads (M)	Accession
O. frankpressi	Full body	gDNA	PacBio	3.28	ERS12522189
O. frankpressi	Full body	gDNA	Illumina	192.24	ERS12522190
O. frankpressi	Body	Total RNA	Illumina	129.96	SRR2017400
O. frankpressi	Roots	Total RNA	Illumina	137.70	SRR2017399
Oasisia alvinae	Trunk	gDNA	PacBio	5.57	ERS12522191
Oasisia alvinae	Trunk	gDNA	Illumina	269.88	ERS12522192
Oasisia alvinae	Crown (replicate 1)	Total RNA	Illumina	41.56	ERS12522193
Oasisia alvinae	Crown (replicate 2)	Total RNA	Illumina	46.72	ERS12522194
Oasisia alvinae	Opisthosoma (replicate 1)	Total RNA	Illumina	47.01	ERS12522195
Oasisia alvinae	Opisthosoma (replicate 2)	Total RNA	Illumina	45.75	ERS12522196
Oasisia alvinae	Trophosome (replicate 1)	Total RNA	Illumina	42.72	ERS12522197
Oasisia alvinae	Trophosome (replicate 2)	Total RNA	Illumina	42.15	ERS12522198
R. pachyptila	Vestimentum	gDNA	PacBio	2.64	ERS12522199
R. pachyptila	Vestimentum	gDNA	Illumina	78.49	ERS12522200
R. pachyptila	Crown	Total RNA	Illumina	45.05	ERS12522201
R. pachyptila	Trunk wall	Total RNA	Illumina	40.45	ERS12522202

Supplementary Table 2. List of microbial genomes sequenced from *O. frankpressi*[a]

Group	Bacterium
Endosymbiont	Oceanospirillales RS1 copy1<br>Oceanospirillales RS1 copy2
Epibiont	Arcobacter<br>Sulfurospirillum<br>Sulfurimonas copy1 - HighCov<br>Sulfurimonas copy2 - LowCov
Free-living	Alphaproteobacteria_rhodospirillales<br>Alphaproteobacteria_kordiimonadales<br>Fusobacteriaceae_Psychrilyobacter<br>Fusibacteraceae_Fusibacter<br>Pseudomonadales_Thioglobaceae<br>Desulfocapsaceae

[a] Assemblies are available at the European Nucleotide Archive under bioproject PRJNA813420.

Supplementary Table 3. BUSCO values of the assemblies and the annotations

Species	Complete	Single	Duplicated	Fragmented	Missing
O. frankpressi assembly	80.1%	79.0%	1.1%	6.4%	13.5%
Oasisia alvinae assembly	96.9%	95.1%	1.8%	1.2%	1.9%
R. pachyptila assembly	95.6%	94.5%	1.1%	1%	3.4%
O. frankpressi annotation	91.6%	90.6%	1%	1.9%	6.5%
Oasisia alvinae annotation	97.9%	96%	1.9%	0.7%	1.4%
R. pachyptila annotation	96.8%	96.4%	0.4%	1%	2.2%

Supplementary Table 4. RepeatMasker annotation for *O. frankpressi*

Class	Subclass	Number of elements	Length occupied	Percentage of sequence
SINEs		0	0 bp	0 %
	ALUs	0	0 bp	0 %
	MIRs	0	0 bp	0 %
LINEs		16554	7470495 bp	2.62 %
	LINE1	1191	262659 bp	0.09 %
	LINE2	8930	4880920 bp	1.71 %
	L3/CR1	102	44952 bp	0.02 %
LTR elements		5792	2589492 bp	0.91 %
	ERVL	0	0 bp	0 %
	ERVL-MaLRs	0	0 bp	0 %
	ERV_classI	3021	728659 bp	0.26 %
	ERV_classII	427	107558 bp	0.04 %
DNA elements		58532	15266934 bp	5.36 %
	hAT-Charlie	0	0 bp	0 %
	TcMar-Tigger	2906	329535 bp	0.12 %
Unclassified		87799	26257536 bp	9.22 %
Total interspersed repeats		/	51584457 bp	18.12 %
Small RNA		320	936986 bp	0.33 %
Satellites		4999	555329 bp	0.20 %
Simple repeats		222433	29821287 bp	10.48 %
Low complexity		17229	1377273 bp	0.48 %
**Total bases masked**		**/**	**82995924 bp**	**29.16 %**

Supplementary Table 5. RepeatMasker annotation for *Oasisia alvinae*

Class	Subclass	Number of elements	Length occupied	Percentage of sequence
SINEs		31813	7301688 bp	0.90 %
	ALUs	0	0 bp	0 %
	MIRs	31351	7254252 bp	0.90 %
LINEs		201908	70921252 bp	8.78 %
	LINE1	6831	898275 bp	0.11 %
	LINE2	67934	23752119 bp	2.94 %
	L3/CR1	26088	10752788 bp	1.33 %
LTR elements		80211	15398036 bp	1.91 %
	ERVL	0	0 bp	0 %
	ERVL-MaLRs	0	0 bp	0 %
	ERV_classI	25807	2596412 bp	0.32 %
	ERV_classII	7459	1192793 bp	0.15 %
DNA elements		186008	56807183 bp	7.03 %
	hAT-Charlie	1757	376153 bp	0.05 %
	TcMar-Tigger	209	30159 bp	0 %
Unclassified		623188	164893139 bp	20.41 %
Total interspersed repeats		/	315321298 bp	39.03 %
Small RNA		12830	3636450 bp	0.45 %
Satellites		27203	5678762 bp	0.70 %
Simple repeats		362245	81655285 bp	10.11 %
Low complexity		10824	905243 bp	0.11 %
**Total bases masked**		**/**	**405156532 bp**	**50.15 %**

Supplementary Table 6: RepeatMasker annotation for *R. pachyptila*

Class	Subclass	Number of elements	Length occupied	Percentage of sequence
SINEs		21320	3026230 bp	0.55 %
	ALUs	0	0 bp	0 %
	MIRs	21320	3026230 bp	0.55 %
LINEs		87276	43599677 bp	7.88 %
	LINE1	11208	1624069 bp	0.29 %
	LINE2	27602	8280880 bp	1.50 %
	L3/CR1	29494	28314275 bp	5.11 %
LTR elements		90618	12941344 bp	2.34 %
	ERVL	0	0 bp	0 %
	ERVL-MaLRs	0	0 bp	0 %
	ERV_classI	10399	881879 bp	0.16 %
	ERV_classII	1250	318412 bp	0.06 %
DNA elements		79305	12117760 bp	2.19 %
	hAT-Charlie	1058	164379 bp	0.03 %
	TcMar-Tigger	0	0 bp	0 %
Unclassified		195756	45050193 bp	8.14 %
Total interspersed repeats		/	116735204 bp	21.09 %
Small RNA		809	524256 bp	0.09 %
Satellites		34434	9644285 bp	1.74 %
Simple repeats		192588	30818651 bp	5.57 %
Low complexity		6510	578920 bp	0.10 %
**Total bases masked**		**/**	**154286816 bp**	**27.87 %**

Supplementary Table 7: Pattern recognition receptors in selected annelids[1]

PRRs	Ofra	Oalv	Rpac	Pech	Lluy	Ofus	Ctel
Lectins							
C-type lectin (CTLs)	8	26	14	47	77	85	52
Fibrinogen-related protein (FREPs)	5	62	62	66	80	92	82
Galectin	0	1	1	2	1	6	4
Peptidoglycan recognition protein (PGRP)	1	14	10	9	18	19	7
Scavenger receptor (SR)	2	5	3	3	3	5	10
Toll-like receptor (TLR)	9	50	35	23	46	75	21
Bactericidal permeability increasing protein (BPIP)	1	14	6	7	8	2	8
Nod-like receptor (NLR)	0	60	16	20	23	0	1

[1]Species abbreviations: Ofra, *O. frankpressi*; Oalv, *Oasisia alvinae*; Rpac, *R. pachyptila*; Pech, *P. echinospica*; Lluy, *L. luymesi*; Ofus, *Owenia fusiformis*; Ctel, *C. teleta*.
