# Supplementary material for: Distinct genomic routes underlie transitions to specialised symbiotic lifestyles in deep-sea annelid worms
Source: Nat Commun. 2023 May 17;14:2814. doi: 10.1038/s41467-023-38521-6 (PMC10192322; doi:10.1038/s41467-023-38521-6)
Supplement: Supplementary file 2 — Reporting Summary [file 41467_2023_38521_MOESM2_ESM.pdf]

nature portfolio

Corresponding author(s): Jose M. Martin-Duran
Lee Henry

Last updated by author(s): Apr 7, 2023

Reporting Summary

Nature Portfolio wishes to improve the reproducibility of the work that we publish. This form provides structure for consistency and transparency in reporting. For further information on Nature Portfolio policies, see our Editorial Policies and the Editorial Policy Checklist.

Statistics

For all statistical analyses, confirm that the following items are present in the figure legend, table legend, main text, or Methods section.

n/a	Confirmed	
☐	☒	The exact sample size (*n*) for each experimental group/condition, given as a discrete number and unit of measurement
☐	☒	A statement on whether measurements were taken from distinct samples or whether the same sample was measured repeatedly
☐	☒	The statistical test(s) used AND whether they are one- or two-sided<br>*Only common tests should be described solely by name; describe more complex techniques in the Methods section.*
☒	☐	A description of all covariates tested
☒	☐	A description of any assumptions or corrections, such as tests of normality and adjustment for multiple comparisons
☐	☒	A full description of the statistical parameters including central tendency (e.g. means) or other basic estimates (e.g. regression coefficient) AND variation (e.g. standard deviation) or associated estimates of uncertainty (e.g. confidence intervals)
☐	☒	For null hypothesis testing, the test statistic (e.g. *F*, *t*, *r*) with confidence intervals, effect sizes, degrees of freedom and *P* value noted<br>*Give P values as exact values whenever suitable.*
☐	☒	For Bayesian analysis, information on the choice of priors and Markov chain Monte Carlo settings
☒	☐	For hierarchical and complex designs, identification of the appropriate level for tests and full reporting of outcomes
☒	☐	Estimates of effect sizes (e.g. Cohen's *d*, Pearson's *r*), indicating how they were calculated

Our web collection on statistics for biologists contains articles on many of the points above.

Software and code

Policy information about availability of computer code

Data collection	SRA tools v2.10.8.
Data analysis	Canu v.1.8, pbgcpp v.1.9.0, FastQC v.0.11.8, Cutadapt v.2.5, BWA v.0.7.17, Pilon v.1.23, BlobTools v.2.1, Purge_Dups v.1.0.1, BUSCO v.3.0.2, QUAST v.5.0.2, KAT v.2.4.2, GenomeScope2, Kraken2 v.2.1.0, Krakentools v.0.1, Metaflye v.2.9, NextPolish v.1.4.0, Bandage v.0.9.0, MaxBin2 v.2.2.7, CheckM v.1.0.8, MetaQuast v.5.2.0, Prokka v.1.14.5, MacSyFinder v.2, BlastKOALA v.2.2, KEGG Mapper v.5, GSEA v.4.2.3, OrthoVenn2 v.2, GTDB-Tk v.1.6.0, Circos v.0.69-9, RepeatModeler v.2.0.1, DIAMOND v.0.8.22, RepeatMasker v.4.1.0, LTR-finder v.1.07, RepeatCraft v.1, TEclass v.2.1.3c, ggplot2 v.3.3.0, Trinity v.2.9.1, Trimmomatic v.0.35, GMAP v.2017.09.30, STAR v.2.7.5a, StringTie v.2.1.2, Portcullis v.1.2.2, Mikado v.2.Orc2, Augustus v.3.3.3, Exonerate v.2.4.0, PASA v.2.4.1, Trinotate v.3.2.1, PANTHER v.1.0.10, KAAS v.2.1, AGAT v.0.5.0, minimap2 v.2.17, cd-hit v.4.8.1, BLAST v.2.12.0+, PFAMscan v.1.6, OrthoFinder v.2.5.2, TopGO v.2.42.0, Kallisto v.0.46.2, pheatmap v.1.0.12, TBtools v.1.042, hmmer-3.1b2, CLANS, MAFFT, trimAl v.1.4.rev15, FastTree v.2.1.10, IQ-Tree v.2.2.0-beta, MrBayes v.3.2.7a, FigTree v.1.4.4, CD-Search v.3.20, python 3.8.5

For manuscripts utilizing custom algorithms or software that are central to the research but not yet described in published literature, software must be made available to editors and reviewers. We strongly encourage code deposition in a community repository (e.g. GitHub). See the Nature Portfolio guidelines for submitting code & software for further information.

Data

Policy information about availability of data

All manuscripts must include a data availability statement. This statement should provide the following information, where applicable:

- Accession codes, unique identifiers, or web links for publicly available datasets
- A description of any restrictions on data availability
- For clinical datasets or third party data, please ensure that the statement adheres to our policy

All sequence data associated with this project are available at the European Nucleotide Archive (project PRJEB55047) [https://www.ncbi.nlm.nih.gov/bioproject/PRJEB55047]. This study also used previously published datasets with accessions SRR2017399 [https://www.ncbi.nlm.nih.gov/sra/SRR2017399], SRR2017400 [https://www.ncbi.nlm.nih.gov/sra/SRR2017400], SRR8949056–SRR8949077 [https://www.ncbi.nlm.nih.gov/bioproject/PRJNA534438]. Additional files are publicly available at https://github.com/ChemaMD/OsedaxGenome. Source data are provided as a Source Data file.

Human research participants

Policy information about studies involving human research participants and Sex and Gender in Research.

Reporting on sex and gender	Not applicable
Population characteristics	Not applicable
Recruitment	Not applicable
Ethics oversight	Not applicable

Note that full information on the approval of the study protocol must also be provided in the manuscript.

Field-specific reporting

Please select the one below that is the best fit for your research. If you are not sure, read the appropriate sections before making your selection.

☐ Life sciences ☐ Behavioural & social sciences ☒ Ecological, evolutionary & environmental sciences

For a reference copy of the document with all sections, see nature.com/documents/nr-reporting-summary-flat.pdf

Ecological, evolutionary & environmental sciences study design

All studies must disclose on these points even when the disclosure is negative.

Study description	Sequencing of the genome of the symbiotic annelids Osedax frankpressi, Oasisia alvinae and Riftia pachyptila
Research sample	Entire individual of Osedax frankpressi, and given their size, tissue dissections of Oasisia alvinae and Riftia pachyptila. In all cases, these were healthy representative adult specimens.
Sampling strategy	Specimens were collected randomly. Genome sequencing was performed from one single individual to avoid heterozygosity.
Data collection	This study does not involve collection of data from the wild. Specimens for genome sequencing were collected with remotely operated vehicles
Timing and spatial scale	Specimens were collected once on the 30th November 2004 (Osedax) and 14th November 2018 (Oasisia and Riftia)
Data exclusions	No data was excluded from any of the analyses
Reproducibility	Two biological replicates were collected for most RNA-seq datasets, whenever possible, which is a commonly accepted standard in the field. All attempts to replicate were successful.
Randomization	Tissue samples were allocated randomly for genomic and transcriptomic sequencing.
Blinding	Specimens were chosen blindly for genome sequencing

Did the study involve field work? ☒ Yes ☐ No

Field work, collection and transport

Field conditions	Specimens were collected at 2000-4000m depth in a whale-fall (Osedax) and a vent site (Oasisia and Riftia)
Location	Whalefall 2893, Monterey Submarine Canyon (36.78°N/122.08°W), California (the US) at a depth of 2893m; Matterhorn vent site (108°51'47"W/23°57'15"N) in the Gulf of California, Mexico, at a depth of 3653.5m
Access & import/export	Mexican samples were collected under CONAPESCA permit PPFE/DGOPA-200/18.
Disturbance	Collection was done with a specialised robot to minimise disturbance of the colonies.

Reporting for specific materials, systems and methods

We require information from authors about some types of materials, experimental systems and methods used in many studies. Here, indicate whether each material, system or method listed is relevant to your study. If you are not sure if a list item applies to your research, read the appropriate section before selecting a response.

Materials & experimental systems

n/a	Involved in the study
☒	☐ Antibodies
☒	☐ Eukaryotic cell lines
☒	☐ Palaeontology and archaeology
☐	☒ Animals and other organisms
☒	☐ Clinical data
☒	☐ Dual use research of concern

Methods

n/a	Involved in the study
☒	☐ ChIP-seq
☒	☐ Flow cytometry
☒	☐ MRI-based neuroimaging

Animals and other research organisms

Policy information about studies involving animals; ARRIVE guidelines recommended for reporting animal research, and Sex and Gender in Research

Laboratory animals	The study did not involve laboratory animals
Wild animals	Osedax frankpressi, Oasisia alvinae and Riftia pachyptila were collected with specialised robots in the deep-sea of California and Mexico. Specimens were brought to surface, dissected and preserved by flash freezing the samples.
Reporting on sex	Sex did not affect and was not considered in our study.
Field-collected samples	No lab work was done on live animals collected from the field.
Ethics oversight	Annelid worms are not regulated by ethical limitations.

Note that full information on the approval of the study protocol must also be provided in the manuscript.
