# Supplementary material for: Distinct genomic routes underlie transitions to specialised symbiotic lifestyles in deep-sea annelid worms
Source: Nat Commun. 2023 May 17;14:2814. doi: 10.1038/s41467-023-38521-6 (PMC10192322; doi:10.1038/s41467-023-38521-6)
Supplement: Supplementary file 4 — Description of Additional Supplementary Files [file 41467_2023_38521_MOESM4_ESM.docx]

Supplementary Data 1 (tab 1). List of manually curated missing BUSCO genes in *O. frankpressi*.

Supplementary Data 2 (tab 2). Species and genomes used for gene family evolutionary analyses.

Supplementary Data 3 (tab 3). Multiplatform annotation of the *Osedax* endosymbiont genome showing all coding sequences tagged with their respective COG category, common gene name, description of protein product, KEGG identifier, PFAM domain tag, and Gene Ontology identifier.

Supplementary Data 4 (tab 4). Multiplatform annotation of the *Oasisia* endosymbiont genome showing all coding sequences tagged with their respective COG category, common gene name, description of protein product, KEGG identifier, PFAM domain tag, and Gene Ontology identifier.

Supplementary Data 5 (tab 5). Proteins present in newly sequenced Oceanospirillales, absent in the previously published Rs1 genome.

Supplementary Data 6 (tab 6). Comparison of *O. frankpressi* endosymbiont with free-living relatives.

Supplementary Data 7 (tab 7). Comparison of *O. frankpressi* endosymbiont with the endosymbiont of Vestimentifera.

Supplementary Data 8 (tab 8). List and taxonomic distribution of Eukaryotic-like domains in the symbionts of Siboglinidae.

Supplementary Data 9 (tab 9). Pathways for carbohydrate and lipid metabolism in animals and their completeness in annelid genomes.

Supplementary Data 10 (tab 10). Biosynthetic capabilities in asymbiotic annelids, siboglinids and the main symbiont of *Osedax frankpressi*.

Supplementary Data 11 (tab 11). Pattern recognition receptors in *O. frankpressi*.

Supplementary Data 12 (tab 12). Pattern recognition receptors in *Oasisia alvinae*.

Supplementary Data 13 (tab 13). Pattern recognition receptors in *R. pachyptila*.

Supplementary Data 14 (tab 14). Pattern recognition receptors in *P. echinospica*.

Supplementary Data 15 (tab 15). Pattern recognition receptors in *L. luymesi*.

Supplementary Data 16 (tab 16). Pattern recognition receptors in *Owenia fusiformis*.

Supplementary Data 17 (tab 17). Pattern recognition receptors in *C. teleta*.

Supplementary Data 18 (tab 18). G protein-coupled receptor repertoires in bilaterians.

Supplementary Data 19 (tab 19). Transcription factor repertoires of *O. frankpressi* and selected annelids.

Supplementary Data 20 (tab 20). DNA repair pathways in *O. frankpressi* and selected annelids.
